# Spodium Bonds Involving Methylmercury and Ethylmercury in Proteins: Insights from X-ray Analysis and Computations

**DOI:** 10.1021/acs.inorgchem.3c02716

**Published:** 2023-10-30

**Authors:** Sergi Burguera, Akshay Kumar Sahu, Antonio Frontera, Himansu S. Biswal, Antonio Bauza

**Affiliations:** † Department of Chemistry, Universitat de les Illes Balears, Ctra. de Valldemossa km 7.5, 07122 Palma, Baleares, Spain; ‡ School of Chemical Sciences, National Institute of Science Education and Research (NISER), Bhubaneswar 752050, India; § Training School Complex, Homi Bhabha National Institute, Mumbai 400094, India

## Abstract

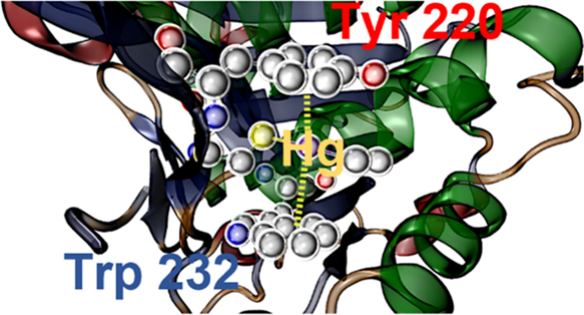

In this study, the stability, directionality, and physical nature of Spodium bonds (SpBs, an attractive noncovalent force involving elements from group 12 and Lewis bases) between methylmercury (MeHg) and ethylmercury (EtHg) and amino acids (AAs) have been analyzed from both a structural (X-ray analysis) and theoretical (RI-MP2/def2-TZVP level of theory) point of view. More in detail, an inspection of the Protein Data Bank (PDB) reported evidence of noncovalent contacts between MeHg and EtHg molecules and electron-rich atoms (e.g., O atoms belonging to the protein backbone and S atoms from MET residues or the π-systems of aromatic AAs such as TYR or TRP). These results were rationalized through a computational study using MeHg coordinated to a thiolate group as a theoretical model and several neutral and charged electron-rich molecules (e.g., benzene, formamide, or chloride). The physical nature of the interaction was analyzed from electrostatics and orbital perspectives by performing molecular electrostatic potential (MEP) and natural bonding orbital (NBO) analyses. Lastly, the noncovalent interactions plot (NCIplot) technique was used to provide a qualitative view of the strength of the Hg SpBs and compare them to other ancillary interactions present in these systems as well as to shed light on the extension of the interaction in real space. We believe that the results derived from our study will be useful to those scientists devoted to protein engineering and bioinorganic chemistry as well as to expanding the current knowledge of SpBs among the chemical biology community.

## Abstract 2

Spodium bonding interactions involving methyl and ethylmercury cations in protein structures were studied at the RI-MP2/def2-TZVP level of theory in a combined Protein Data Bank and computational study.

## Introduction

Methylmercury (MeHg), an organometallic compound derived from inorganic mercury (iHg) through a process called methylation,^1^ is commonly synthesized in aquatic environments by certain bacteria and archaea microorganisms.^2−5^ This leads to a bioaccumulation and biomagnification of MeHg through the food chain, resulting in its increased concentration in higher trophic levels, including fish and seafood, which are common dietary sources for humans.^6,7^ MeHg exhibits particular chemical features that facilitate crossing biological barriers, such as the blood–brain barrier,^8−11^ thus exerting a broad spectrum of toxicological effects on the nervous system,^12^ which ultimately lead to cognitive deficits, developmental delays, and neurological disorders. Additionally, recent studies suggest that MeHg exposure may also contribute to neurodegenerative diseases, such as Alzheimer’s and Parkinson’s.^13−15^ Beyond its neurotoxic effects, MeHg can also impact other organ systems, such as kidney function,^16^ interfere with the cardiovascular system,^17^ disrupt the endocrine system,^18^ and weaken the immune response.^19^ Its environmental impact cannot be underestimated either, since MeHg can also influence ecosystems by altering the behavior, reproduction, and survival of various organisms, thus impacting the overall biodiversity.^20−22^

From a molecular biology perspective, MeHg acts as a disruptive agent of noncovalent interactions crucial for protein folding, stability, and function.^23,24^ It can bind to thiolate groups in cysteine residues, leading to conformational changes and the misfolding of proteins. In addition, it can affect DNA structure and function through the binding to DNA bases and phosphate backbone, thus provoking DNA damage, strand breaks, and interference with DNA replication and repair mechanisms.^25,26^ Hence, advancing in understanding the role of MeHg in biological systems is crucial for understanding its impact, assessing risks, and developing strategies for its mitigation.

In this regard, although the molecular anchoring of MeHg in a protein’s cavity is based on the coordination to a CYS residue, the vicinal amino acids (AAs) also need to alter their disposition and disrupt their native network of noncovalent interactions (NCIs) to accommodate MeHg. The term Spodium bond (SpB)^27^ was recently proposed to classify the NCIs involving group 12 of elements (Zn, Cd, and Hg) when acting as Lewis acids. The biological implications of this novel noncovalent force have been explored by some of us in the case of Zn,^28^ thus expanding its structural and functional role in biology. SpBs are part of the “σ-hole chemistry”, which involves NCIs from the p-block^29−33^ and, more recently, from the d-block of elements, such as Wolfium (group 6),^34^ Matere (group 7),^35^ Osme (group 8),^36^ and Regium bonds (group 11).^37,38^ These imply electrophilic regions located on the Lewis acid molecule (usually characterized by a positive electrostatic potential) that favorably interact with a Lewis base (e.g., a lone pair, a π-system, or an anion).^39^

In this study, our approach consisted on a combination of a Protein Data Bank (PDB)^40^ survey and an *ab initio* theoretical study at the RI-MP2/def2-TZVP level of theory to analyze the NCIs responsible for the stabilization of MeHg and ethylmercury (EtHg) (another toxic Hg methylation derivative) in biological systems. To achieve that, an inspection of the PDB revealed 586 contacts involving MeHg and EtHg coordinated to CYS residues and electron-rich atoms (N, O, or S). Using theoretical models, we evaluated the stability and directionality of several selected Hg SpBs gathered from the PDB search. These results were complemented with an *ab initio* computational study at the RI-MP2/def2-TZVP level of theory. The noncovalent nature of the interaction was assessed using the quantum theory of atoms in molecules (QTAIM), the natural bonding orbital (NBO), and the noncovalent interactions plot (NCIplot) analyses. We believe the results reported herein (i) will assist in increasing the visibility of SpBs among the bioinorganic chemistry community and (ii) provide new insights into the NCIs responsible for the stabilization of MeHg and EtHg in proteins, which might have great impact in the fields of chemical biology and environmental chemistry.

## Methods

The RCSB website was accessed in May 2023 to download PDB files containing mercury (Hg), resulting in 694 files with at least one Hg atom at a resolution of up to 4 Å. These were filtered out for methyl or ethylmercury-containing structures, leaving 34 PDB files for further analysis. Our analysis focused on two main criteria. First, we identified the C–Hg–S moiety by examining the 2.5 Å vicinity around the Hg atom to determine sulfur (S) atom binding. Subsequently, we searched for Hg···A SpBs, where A represented nitrogen (N), oxygen (O), or sulfur (S) atoms. The geometric criterion for Hg···A distance was set between 2.5 and 4.5 Å and the minimum distance was kept at 2.5 Å to avoid the overlap between coordination and Spodium bonding interactions. The entire analysis was performed by using a custom Python code developed by us.

### Computation of the SpB Energies in Selected PDB Structures

Once the PDB structures were identified, theoretical models were built containing MeHg/EtHg molecules coordinated to a thiolate group (CH_3_–Hg–SCH_3_) and the interacting amino acid (composed by the side chain and the backbone atoms, see the Supporting Information for the Cartesian coordinates of the PDB models used). In a later stage, the H atoms from the PDB models were optimized at the PBE0^41,42^-D3^43^/def2-SVP^44^ level of theory. These optimized geometries were then taken as a starting point for single-point calculations at the RI-MP2/def2-TZVP level of theory to compute the interaction energies given in Table 1. The interaction energies were corrected by using the Boys and Bernardi basis set superposition error (BSSE) counterpoise technique.^45^

**Table 1:** List of PDB Codes Retrieved from the Search (PDB ID) Including the Interacting Partners (SpB Donor and AA), the Resolution of the Structure (in Å), the BSSE-Corrected Energies (Δ*E*_BSSE_, in kcal/mol), Geometrical Parameters (Distance *d*_A···Hg_, in Å and A···Hg–C Angle, in Degree), and the Value of the Density at the BCP That Characterizes the Hg SpBs (ρ × 10^2^) at the RI-MP2/def2-TZVP Level of Theory

PDB ID	SpB donor	AA	resolution	Δ*E*_BSSE_	*d*_A···Hg_	A···Hg–C	ρ × 10^2^
1EMS	EtHg	HIS98 (C)	2.8	–4.5	2.976	91.9	1.79
1IRK	EtHg	MET1109 (S)	2.1	–2.6	3.264	72.8	1.50
1RHY	EtHg	SER142 (O)	2.3	–2.1	3.446	112.3	0.57
1X8K	EtHg	ALA316 (O)	2.8	–2.7	3.951	94.4	0.22
3PYK	MeHg	GLN137 (O)	1.3	–5.3	3.041	92.2	1.27
5LU8	EtHg	TYR220 (C)	1.9	–5.4	3.866	92.0	0.54
		TRP232 (C)		–10.0	3.846	76.6	0.43
6BZI	EtHg	THR375 (O)	2.4	–5.2	2.951	73.9	1.54
		GLU448 (O)		–12.4	3.380	110.2	0.63
6PII	EtHg	GLN217 (O)	1.9	–5.1	2.868	75.1	1.68
1L9A	MeHg	TYR68	2.9	–1.9	3.489	65.2	a
2D2N	MeHg	PHE63	3.2	–5.6	3.084	70.2	1.57
5G5N	MeHg	CYS219	2.3	–6.3	2.993	92.7	1.35

a In this complex, no A···Hg BCP was found.

### Computation of the Spodium Bond Energies Using Fully Optimized Models (Complexes **1**–**17**)

The interaction energies of all complexes were computed at the RI-MP2^46^/def2-TZVP^44^ level of theory (also corrected using the BSSE counterpoise technique), which is adequate for the treatment of NCIs involving neutral and charged electron donors.^47^ The calculations have been performed using the program TURBOMOLE version 7.0.^48^ by fully optimizing the geometries without imposing any restraints. Only the Cs symmetry point group was imposed during the optimization process (except for complex 14). The interaction energies were calculated using the supermolecule approximation (Δ*E*_SpB_ = *E*_SpB complex_ – *E*_monomer1_ – *E*_monomer2_).

The MEP (molecular electrostatic potential) surfaces were computed at the RI-MP2/def2-TZVP level of theory by means of the TURBOMOLE 7.0 program and were analyzed using Multiwfn software^49^ and visualized using the Gaussview 5.0 program.^50^ The calculations for the wave function analysis^51^ have been carried out at the RI-MP2/def2-TZVP level of theory also using Multiwfn software. The NBO^52^ analyses was performed at the HF/def2-TZVP level of theory by means of the NBO 7.0 program.^53^ Lastly, the NCIplot^54^ isosurfaces correspond to both favorable and unfavorable interactions, as differentiated by the sign of the second density Hessian eigenvalue and defined by the isosurface color. The color scheme is a red–yellow–green–blue scale with red for repulsive (ρ_cut_^+^) and blue for attractive (ρ_cut_^–^) NCI interaction density. Yellow and green surfaces correspond to weak repulsive and attractive interactions, respectively. The VMD^55^ program was used in the visualization of the results from the QTAIM, NBO, and NCIplot analyses.

## Results and Discussion

### PDB Analysis

**Figure 1:**
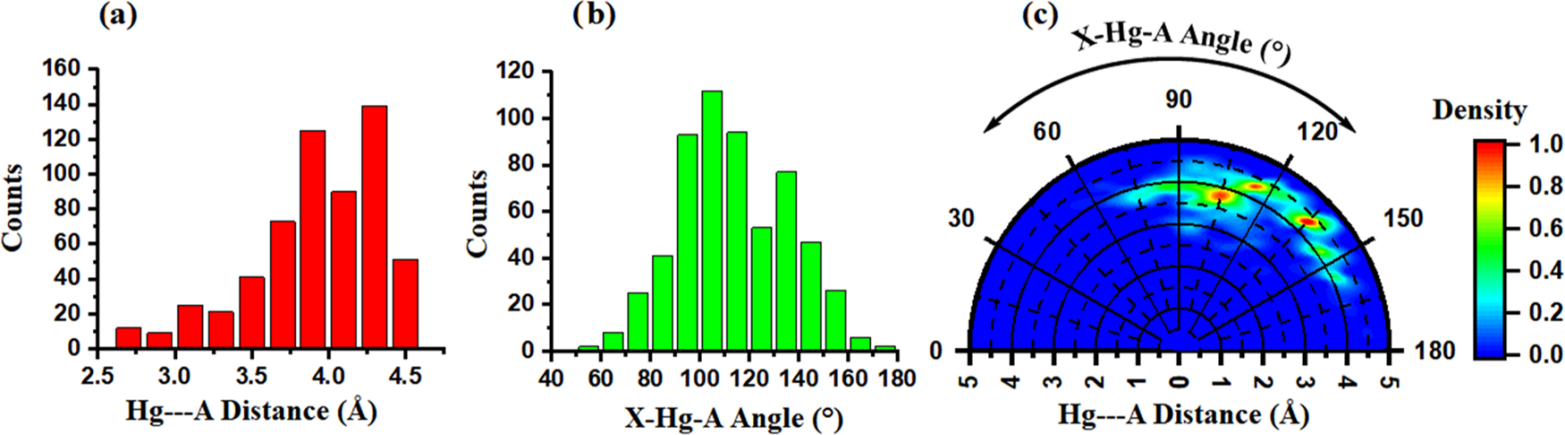
(a) Distance distribution of Hg···A contacts, (b) X–Hg–A angle distribution (X = C, S), and (c) radial distribution plot between Hg···A distance and X–Hg···A angle. The density scale is normalized with respect to the maximum count, where red represents maximum counts and blue represents minimum counts.

Out of the 34 PDB structures containing methyl or ethylmercury, the Hg atoms of 20 structures were coordinated to the cysteine sulfur atom. These 20 structures yielded 586 Spodium bond contacts that satisfied the imposed geometrical criteria. Further analysis of these contacts was conducted, as shown in Figure 1. In Figure 1a, the Hg···A distance distribution demonstrates that the number of contacts increases with distance, as expected due to the increase in volume around the Hg atom and the corresponding higher probability of the presence of another atom. Additionally, the presence of two peaks near 4.0 and 4.25 Å suggests the existence of SpBs centered around the Hg atom. The X–Hg–A angle distribution, where X represents S or C atoms, is shown in Figure 1b. The distribution reveals a peak centered at around 110°, indicating that the perpendicular area to the X–Hg···A plane experiences less steric crowding. These less crowded areas are known to be favorable for the formation of noncovalent interactions, as denoted in the radial distribution plot depicted in Figure 1c. Concretely, the plot reveals two high-density regions centered around 4.0 Å and 110° as well as 4.25 Å and 130°, representing the distance (Hg···A) and angle (X–Hg···A), respectively.

### Selected PDB Examples

**Figure 2:**
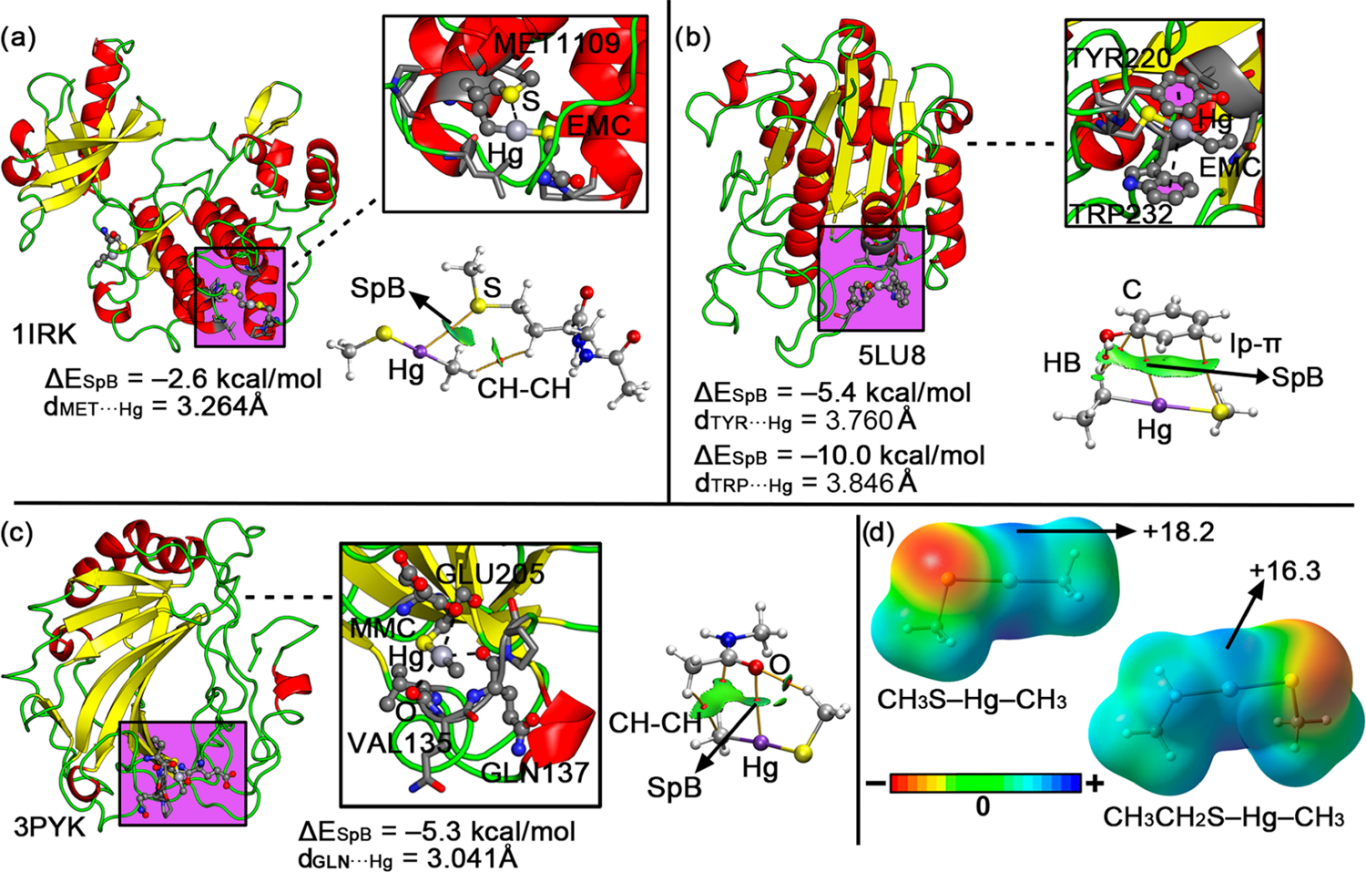
Spodium bonds (SpBs) in (a) 1IRK, (b) 5LU8, and (c) 3PYK structures. The interactions are magnified inside the square parts of the figure, also including QTAIM and NBO analyses of each SpB complex. In panel (d), the MEP surfaces of the MeHg and EtHg molecules are shown (energy values in kcal/mol at 0.001 au). Distances are measured as the shortest value between the Hg atom and the interacting AA.

With the purpose to analyze in more detail the Hg···A (A = N, O, and S) contacts retrieved from the PDB search, three X-ray structures were chosen for computations at the RI-MP2/def2-TZVP level of theory (see Figure 2). First, structure 1IRK(56) (Figure 2a) exhibits a Hg···S SpB (*d* = 3.264 Å) involving an EtHg molecule and a vicinal methionine residue (MET1109), likely acting as a bridge between two α helices from the tyrosine kinase domain of the human insulin receptor protein. Second, structure 5LU8(57) (Figure 2b) belonging to the human legumain protein exhibits a “sandwiched” C_TYR_···Hg···C_TRP_ SpB (*d*_TYR_ = 3.760 Å and *d*_TRP_ = 3.846 Å) involving two aromatic residues (TYR220 and TRP232) located on a protein loop and an EtHg molecule. Lastly, in the 3PYK(58) structure (Figure 2c) involving the human carbonic anhydrase II, three simultaneous Hg···O SpBs were undertaken involving VAL135, GLU205, and GLN137 backbone carbonyl groups and a MeHg moiety (*d*_O-VAL_ = 3.361 Å, *d*_O-GLU_ = 3.312 Å, and *d*_O-GLN_ = 3.041 Å).

In order to rationalize the nature of these interactions from an electrostatics point of view, we also computed the electrostatic potential surfaces of the alkyl Hg derivatives (Figure 2d), showing an electropositive belt around the Hg atoms with a similar electrostatic potential value (+18.2 and +16.3 kcal/mol for MeHg and EtHg, respectively) in line with that obtained for other linear transition-metal coordination complexes.^59,60^ It is also important to note that although the common disposition of an SpB implies a σ-hole^27,28^ (involving an antibonding metal–ligand orbital), the lineal geometry observed in the Hg coordination complexes studied herein precluded the presence of a σ-hole, thus resulting in the electropositive belt observed around the Hg atom (resembling a π-hole).

Furthermore, we also computed the QTAIM and NCIplot analyses of the noncovalent complexes present in these three structures, and the results show a bond critical point (BCP) connecting the lone pair donor atom (S and O) or the π-system from the AA to the Hg atom from the MeHg/EtHg moiety, thus characterizing the SpB. In addition, ancillary CH···CH, lone pair–π (lp–π), and hydrogen bond (HB) interactions were also present (as denoted by their corresponding BCPs and bond paths), also contributing to the binding affinities obtained. Finally, the NCIplot analyses accounted for the weak nature and extension in real space of the interaction, as is deduced from the greenish isosurfaces observed between both counterparts.

In Table 1, the interaction energies, geometrical parameters (including interaction distances and angles), and values of the density at the BCP that connects the Hg atom with the electron-rich moiety are shown for a series of selected X-ray structures gathered from the PDB search. These were selected based on the X-ray resolution, the alkylated Hg moiety involved, and the interaction distance and angle values in order to provide a representative view of the interaction. As noted, the energy values obtained are far from a coordination bond energy (ranging between −10.0 and −1.9 kcal/mol) with Hg···A distances comprised between 2.8 and 4.0 Å.

### Energetic Study

**Figure 3:**
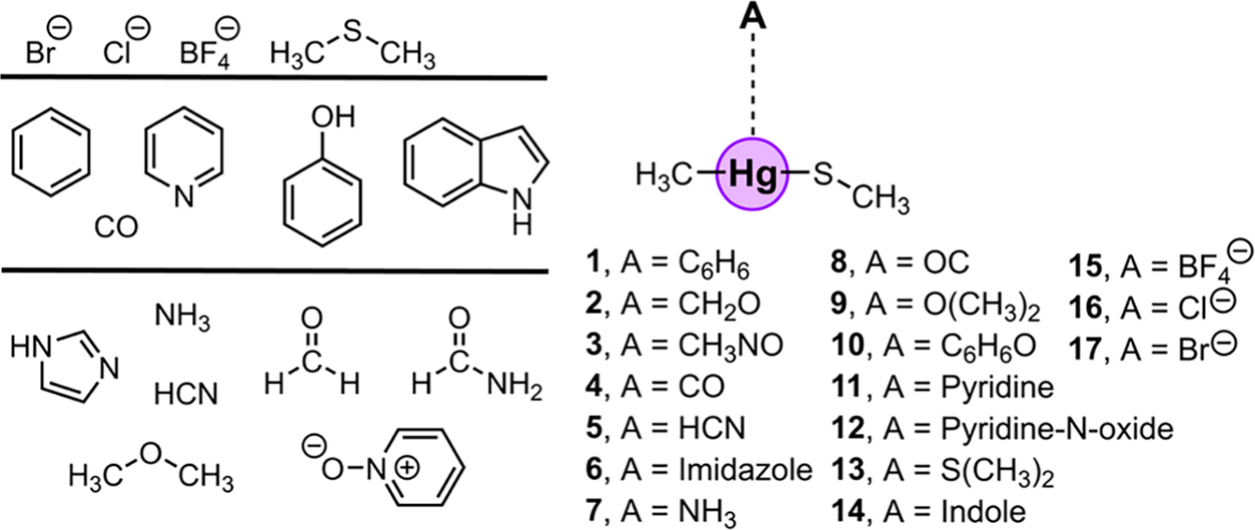
Schematic representation of the compounds and complexes used in this study.

To get further insights into the Hg SpBs present in these systems, we designed a computational study at the RI-MP2/def2-TZVP level of theory using a set of electron-rich species and a MeHg molecule coordinated to a thiolate group (Figure 3) as theoretical models. The energetic results are shown in Table 2, and from their inspection, several interesting points arise.

**Table 2:** BSSE-Corrected Interaction Energies (Δ*E*_BSSE_, in kcal/mol), Geometrical Parameters (Distance *D*_A···Hg_, in Å and A···Hg–C Angle, in Degree), and the Value of the Density at the Bond Critical Point (ρ × 10^2^) Involving the SpB and the Ancillary Interactions Present in Complexes **1**–**17** at the RI-MP2/def2-TZVP Level of Theory

complex	Δ*E*_BSSE_	*D*_A···Hg_	A···Hg–C	ρ × 10^2^ (SpB)	ρ × 10^2^ (ancillary)
**1**	–6.5	3.191	108.9	1.26	0.85 (HB)/0.77 (CH–π)
**2**	–4.4	2.967	95.4	1.52	0.80 (HB)
**3**	–7.9	2.821	93.7	1.96	1.40 (HB)
**4**	–2.0	3.422	109.5	0.77	a
**5**	–4.2	3.191	98.1	0.98	0.78 (lp–π)
**6**	–8.0	2.747	97.9	2.79	0.81 (HB)
**7**	–5.2	2.884	102.8	2.20	a
**8**	–0.9	3.420	107.1	0.51	a
**9**	–4.8	2.841	97.2	2.05	0.54 (HB)/0.54 (HB)
**10**	–9.6	3.277^b^	94.9^b^	1.93	1.80 (HB)/0.71 (CH–π)
**11**	–7.1	2.797	98.1	2.60	0.94 (HB)/0.51 (HB)
**12**	–7.2	2.769	87.1	2.15	1.13 (HB)
**13**	–5.8	3.230	98.9	1.88	0.75 (HB)/0.75 (HB)
**14**	–11.4	3.007	96.9	1.77	1.06 (HB)/0.61 (CH–π)
**15**	–9.5	2.680	92.2	2.23	1.02 (HB)
**16**	–23.1	2.674	98.2	4.65	a
**17**	–19.6	2.858	97.6	3.84	a

a In this complex, no BCP was found.

b Distance and angle measured from the ring centroid.

First, in all cases, attractive interaction energies were obtained (between −23.1 and −0.9 kcal/mol), spread between weak (complex **8**) and moderately strong values (complex **16**). Also, the equilibrium distances obtained ranged between 2 and 3.5 Å, in line with the selected structures from the PDB search. As expected, those complexes involving Br^–^ and Cl^–^ (**16** and **17**) obtained the largest interaction energy values (−23.1 and −19.6 kcal/mol, respectively) in the study. In addition, complex **15** involving BF_4_^–^ as the electron donor moiety achieved a less favorable interaction energy value due to its lower basicity compared to the monatomic anionic species.

Among the neutral complexes (**1**–**14**), complex **14** involving an indole ring obtained the most favorable SpB energy (−11.4 kcal/mol) owing to the simultaneous establishment of an SpB and an HB with the MeHg molecule (see the QTAIM and NCIplot Analyses section). On the other hand, complex **8** involving the weakest Lewis base (OC) achieved the poorest interaction energy value of the set (−0.9 kcal/mol). Among the π-system donors used (benzene and phenol), complex **10** involving the latter obtained a more favorable interaction energy value (−9.6 kcal/mol) due to (i) its higher π-basicity and (ii) the formation of an ancillary S···HO HB vs a S···CH HB in complex **1** (see Figure S1 for their respective QTAIM and NCIplot analyses).

Also, for the N-donating species (imidazole, HCN, NH_3_, and pyridine; complexes **5** to **7** and **11**), complexes **6** and **11** involving imidazole and pyridine achieved the most favorable interaction energy values (−8.0 and −7.1 kcal/mol, respectively), although being weaker Lewis bases than NH_3_. This is due to the formation of ancillary HBs involving NH and CH groups from the imidazole and pyridine rings, respectively, and the thiolate group coordinated to MeHg (see Figure S1 for more details). On the other hand, complex **5** involving HCN as electron donor species obtained the lowest energy of this set (−4.2 kcal/mol), as expected.

Finally, among the O donor molecules (formaldehyde, formamide, dimethyl ether, and pyridine-*N*-oxide), complexes **3** and **12** involving formamide and pyridine-*N*-oxide obtained the largest interaction energy values (−7.9 and −7.2 kcal/mol, respectively). This was an unexpected result in the case of complex **3**; however, a strong HB involving the NH_2_ group of formamide and the S atom from the Hg moiety was undertaken, thus noticeably contributing to the total stabilization of this supramolecular complex. On the other hand, in complex **12** involving pyridine-*N*-oxide, this HB involved a CH group, being weaker than that in complex **3** (see the QTAIM and NCIplot Analyses section).

### QTAIM and NCIplot Analyses

**Figure 4:**
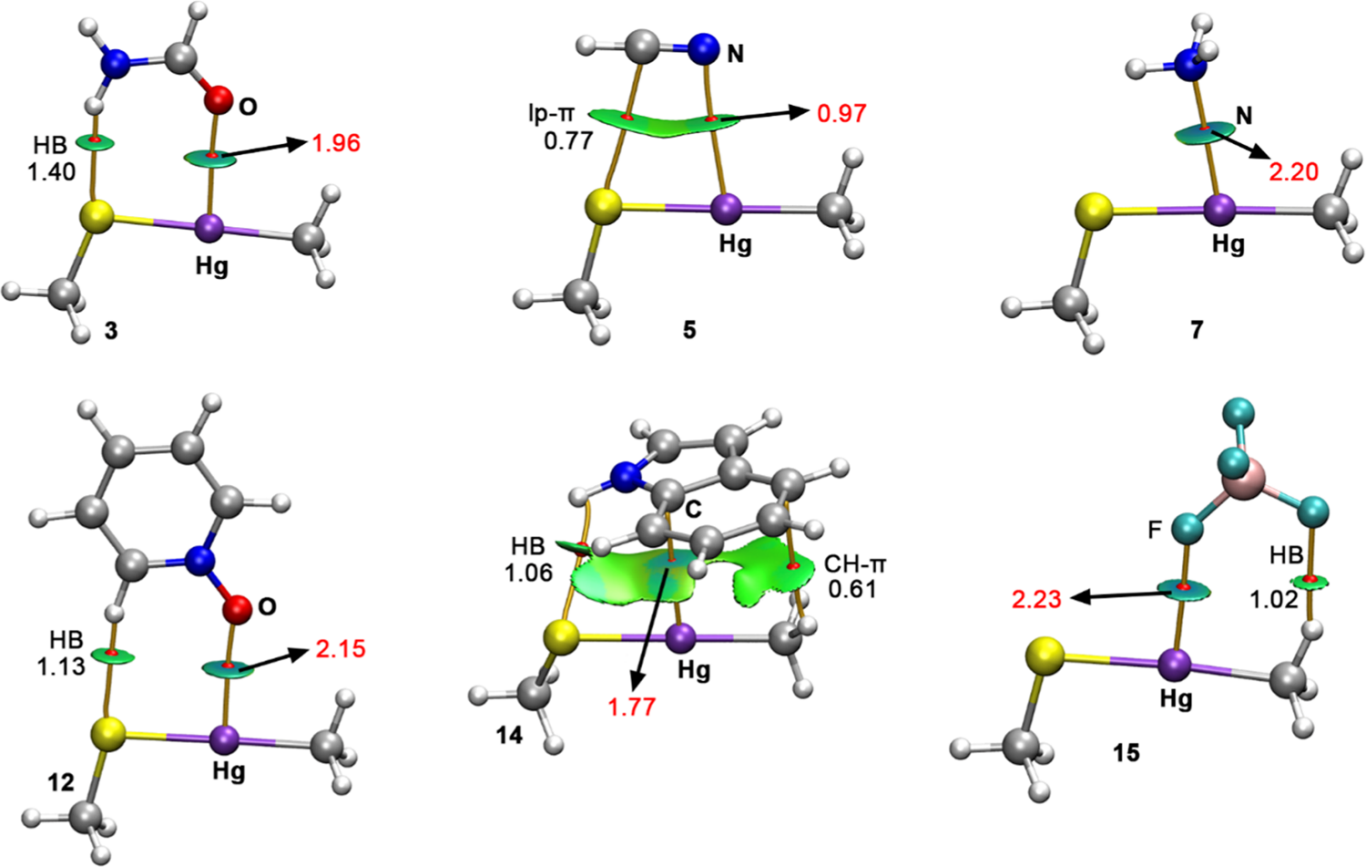
NCIplot analysis and QTAIM distribution of intermolecular bond critical points (BCP in red spheres) and bond paths in complexes **3**, **5**, **7**, **12**, **14**, and **15**. The value of the density at the BCPs characterizing the SpB interaction is also indicated in red. Ancillary interactions with their respective BCP density values are also included. NCIplot surfaces involve only intermolecular contacts between the Sp coordination complex and the electron donor molecule. NCIplot color range −0.04 au ≤ (signλ_2_)ρ ≤ + 0.04 au. Isosurface value RGD = 0.5 and ρ cutoff 0.04 au.

In Figure 4, the combined QTAIM and NCIplot analyses for some representative complexes are shown (the rest are included in Figure S1), and in all cases, a BCP (red sphere) and a bond path connecting the electron donor and Hg atoms were observed, which characterized the Spodium bonding interactions studied herein. Also, ancillary HBs (in complexes **3**, **12**, **14** and **15**) and lp–π (complex **5**) interactions were observed. For instance, in complexes **3**, **12**, and **14**, the HBs involved the NH and CH groups from the formamide and indole moieties and the lone pairs of the S atom coordinated to the Hg center. On the other hand, in the case of complex **12**, the lp–π interaction implied a lone pair from the S atom and the π-system of the HCN molecule. Lastly, in complex **15**, an ancillary HB interaction was described by the presence of a BCP connecting a F atom from the BF_4_^–^ moiety and a CH group from the Hg coordination complex. Interestingly, the value of the density at the BCP that characterizes the SpB interaction exhibits a larger magnitude than that for the ancillary HB and lp–π interactions, thus highlighting the directing role of the Hg SpBs as the predominant noncovalent force in the supramolecular complexes studied herein (see Table 2 for the complete list of ρ × 10^2^ values).

In Table 3, the values of the Laplacian at the BCP that characterize the Hg SpB (∇^2^ρ × 100) are shown, resulting in positive values in all cases, as is common in closed shell calculations. Furthermore, the values of the potential (*V* × 100) and kinetic (*G* × 100) energy densities lie within the same range in all cases, thus confirming the noncovalent nature of the A···Hg interaction (|Vr|/Gr) ≈ 1.

**Table 3:** Values of the Laplacian of ρ (∇^2^ρ × 10^2^, in au), the Potential (*V* × 10^2^, in au) and Kinetic (*G* × 10^2^, in au) Energy Densities, and the Total Energy Density (*H* × 10^2^, in au) Gathered at the BCP That Characterizes the Hg SpB

complex	∇^2^ρ × 10^2^	*V* × 10^2^	*G* × 10^2^	*H* × 10^2^
**1**	3.67	–0.79	0.86	0.06
**2**	5.80	–1.17	1.31	0.14
**3**	7.86	–1.70	1.83	0.13
**4**	2.48	–0.42	0.52	0.10
**5**	3.58	–0.64	0.77	0.13
**6**	9.31	–2.39	2.36	–0.03
**7**	6.94	–1.73	1.74	0.00
**8**	2.14	–0.28	0.41	0.13
**9**	7.66	–1.74	1.83	0.09
**10**	4.69	–0.79	0.98	0.19
**11**	8.34	–2.14	2.11	–0.03
**12**	8.70	–1.94	2.06	0.12
**13**	4.85	–1.23	1.22	–0.01
**14**	5.12	–1.26	1.27	0.01
**15**	10.73	–2.22	2.45	0.23
**16**	14.11	–4.61	4.07	–0.54
**17**	10.01	–3.22	2.86	–0.36

Regarding the NCIplot analyses, in all of the cases, a greenish isosurface was obtained between the electron donor molecule and the Hg moiety, thus indicating the presence of weak interactions. Also worth noting is the fact that the portion of the NCIplot isosurface devoted to the Hg SpB is bluish instead of greenish in the case of complexes **3**, **7**, and **15**, thus indicating that the SpB interaction is noticeably stronger than the ancillary HBs, in line with the results obtained from QTAIM analyses. Finally, in the case of complexes **5** and **14**, all surfaces exhibited a similar color, thus indicating a similar contribution of the ancillary HB and lp–π interactions and the Hg SpBs (see Figure S2 for the plots regarding the reduced density gradient (RDG) vs the sign(λ_2_)ρ).

### NBO Analysis

**Figure 5:**
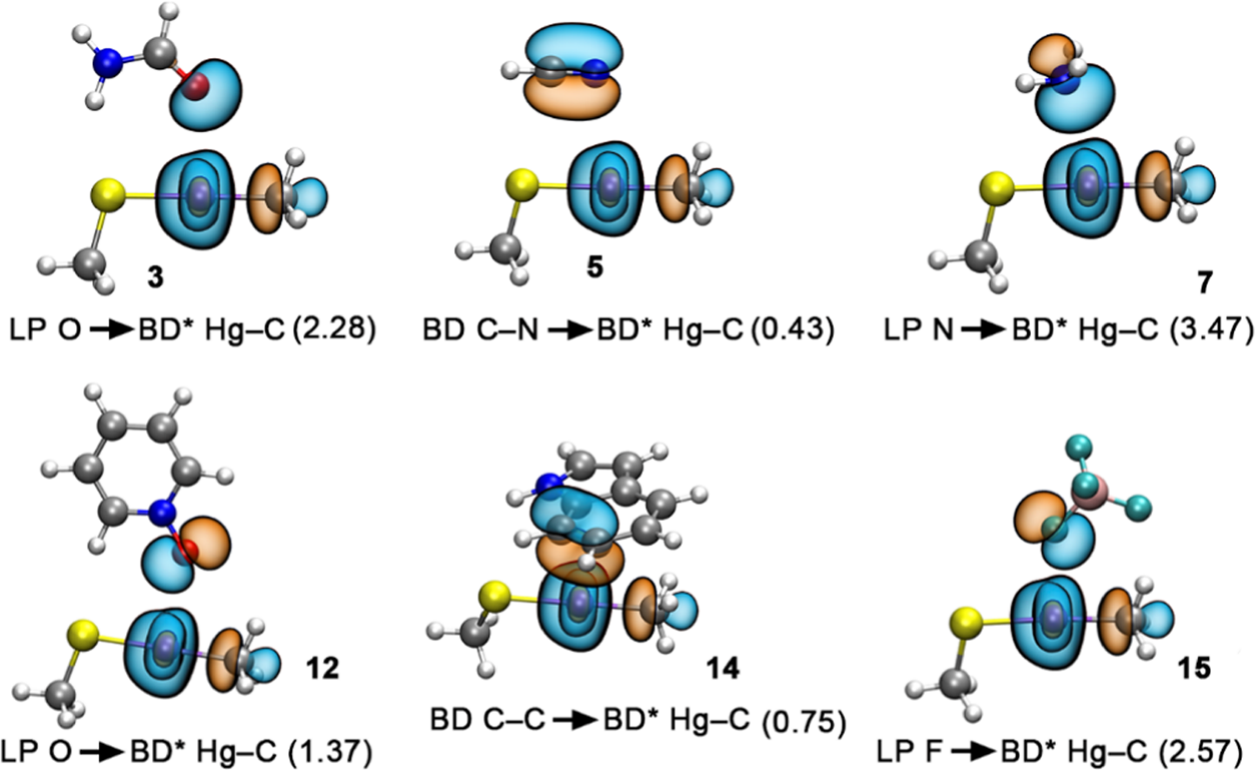
Graphical representation of the donor–acceptor orbitals involved in the formation of complexes **3**, **5**, **7**, **12**, **14**, and **15**. LP, BD, and BD* stand for lone pair, bonding orbital, and antibonding orbital, respectively. Energy values are in kcal/mol.

To further investigate the participation of orbital contributions in the stabilization of the noncovalent complexes studied, we carried out NBO calculations focusing on the second-order perturbation analysis, which is useful to evaluate donor–acceptor interactions (see Table 4). Among the neutral complexes (**1** to **14**), the Hg SpBs were characterized by the interaction between either a lone pair (LP) from an O, S, N, or F atom or a bonding (BD) C–C/N–C orbital belonging to the electron-rich species and an antibonding (BD*) Hg–C orbital from the MeHg moiety. The magnitude of these orbital interactions ranges from 0.19 kcal/mol in complex **8** involving OC as an electron donor molecule to 4.88 kcal/mol in complex **6** involving an imidazole ring. On the other hand, in the case of the anionic complexes (**15**–**17**), the orbital contribution is larger in the case of the monatomic anions Cl^–^ and Br^–^, with values of 18.30 and 16.90 kcal/mol, in line with their respective interaction energies. In addition, in complexes **1** to **3**, **6**, and **12** to **15**, ancillary lp–π, HB, and CH–π interactions were also observed, involving (i) an LP from a S atom and an antibonding (BD*) C–C orbital, (ii) an LP from a S atom and an antibonding (BD*) C–H and N–H orbital, (iii) an LP from a F atom and an antibonding (BD*) C–H orbital, and (iv) a bonding (BD) C–C orbital and an antibonding (BD*) C–H orbital. In all of these cases, the magnitude of the orbital interaction is lower than that observed for the Hg SpB, in line with the results derived from the QTAIM and NCIplot Analyses section (Figure 5).

**Table 4:** Donor and Acceptor NBOs with an Indication of the Second-Order Interaction Energy *E*^(2)^ in Complexes **1**–**17**^a^

complex	type of interaction	donor	acceptor	*E*^(2)^
**1**	SpB	BD C–C	BD* Hg–C	1.45
	lp–π	LP S	BD* C–C	0.67
**2**	SpB	LP O	BD* Hg–C	0.74
	HB	LP S	BD* C–H	0.63
**3**	SpB	LP O	BD* Hg–C	2.28
	HB	LP S	BD* N–H	3.76
**4**	SpB	LP C	BD* Hg–C	0.89
**5**	SpB	BD N–C	BD* Hg–C	0.43
**6**	SpB	LP N	BD* Hg–C	4.88
	HB	LP S	BD* C–H	0.58
**7**	SpB	LP N	BD* Hg–C	3.47
**8**	SpB	LP O	BD* Hg–C	0.19
**9**	SpB	LP O	BD* Hg–C	0.77
**10**	SpB	BD C–C	BD* Hg–C	0.86
**11**	SpB	LP N	BD* Hg–C	4.17
**12**	SpB	LP O	BD* Hg–C	1.37
	HB	LP S	BD* C–H	2.28
**13**	SpB	LP S	BD* Hg–C	3.57
	HB	LP S	BD* C–H	0.62
**14**	SpB	BD C–C	BD* Hg–C	1.41
	HB	LP S	BD* N–H	1.11
	CH–π	BD C–C	BD* C–H	0.24
**15**	SpB	LP F	BD* Hg–C	2.57
	HB	LP F	BD* C–H	0.75
**16**	SpB	LP Cl	BD* Hg–C	18.30
**17**	SpB	LP Br	BD* Hg–C	16.90

a LP, BD, and BD* stand for lone pair, bonding orbital, and antibonding orbital, respectively. Energy values are in kcal/mol.

## Conclusions

In conclusion, we demonstrated the presence of Hg SpBs involving MeHg and EtHg in proteins using a combination of X-ray analysis and theoretical calculations at the RI-MP2/def2-TZVP level of theory. The PDB survey revealed a preference of the SpB at 4.0 and 110° as well as 4.25 and 130°, representing the distance (Hg···A) and angle (X–Hg···A), respectively. This is in alignment with the expected less hindered region in the X–Hg···A plane. Besides, a variety of electron donor molecules was used to analyze the physical nature and extension in real space of the Hg SpB interaction (including O, S, and π-systems from aromatic residues) in a computational study. These computations were complemented with QTAIM and NCIplot analyses, which are utilized to further understand the weak nature of the interaction from a charge–density perspective as well as with NBO analyses, which highlighted the main orbital contributions responsible for the stabilization of the SpBs studied herein. We expect that the results derived from our study will be useful to those scientists devoted to protein engineering and bioinorganic chemistry as well as to expand the current knowledge of the SpBs among the chemical biology community.

## Supporting Information Available

Figure S1 including additional QTAIM and NCIplot analyses and Figure S2 showing the NCI plots of reduced gradient vs sign(λ_2_)ρ and Cartesian coordinates of complexes **1**–**17** and of the PDB models used (PDF)
