# Supplementary material for: Spodium Bonds Involving Methylmercury and Ethylmercury in Proteins: Insights from X-ray Analysis and Computations
Source: Inorg Chem. 2023 Oct 30;62(45):18524–32. doi: 10.1021/acs.inorgchem.3c02716 (PMC10647129; doi:10.1021/acs.inorgchem.3c02716)
Supplement: Supplementary file 1 — ic3c02716_si_001.pdf [file ic3c02716_si_001.pdf]

Electronic Supporting Information

Spodium Bonds involving methylmercury and ethylmercury in proteins: Insights from X-ray analysis and computations.

Sergi Burguera,[a] Akshay Kumar Sahu,[b] Antonio Frontera,[a] Himansu S. Biswal,*,[b] and Antonio Bauzá*,[a]

[a]Department of Chemistry, Universitat de les Illes Balears, Crta. de Valldemossa km 7.5, 07122 Palma (Baleares), SPAIN; Fax: (+) 34 971 173426; E-mail: antonio.bauza@uib.es

[b]School of Chemical Sciences, National Institute of Science Education and Research (NISER), 752050 Bhubaneswar, India; Training School Complex, Homi Bhabha National Institute, 400094 Mumbai, India; E-mail: himansu@niser.ac.in

Figure S1 Page S2

Figure S2 Page S2

Cartesian Coordinates of Complexes 1 to 17 Page S3

Cartesian coordinates of the selected PDB structures Page S9

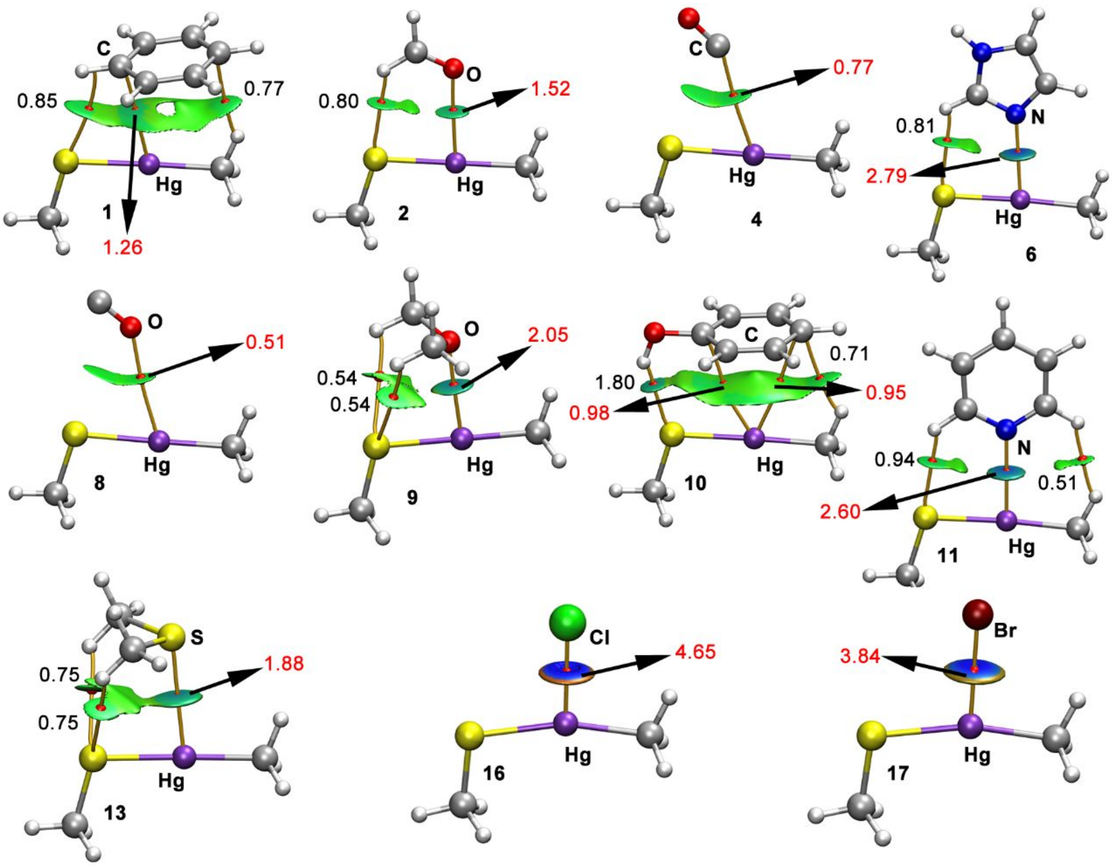

Figure S1. NCIplot analysis and AIM distribution of intermolecular bond critical points (BCP in red spheres) and bond paths in complexes **1**, **2**, **4**, **6**, **8**-**11**, **13**, **16** and **17**. The value of density at the BCPs characterizing the SpB interaction is also indicated in red. Ancillary interactions with their respective BCP density values are also included. NCIplot surfaces involving only intermolecular contacts between the Sp coordination complex and the electron donor molecule. NCIplot color range −0.035 au ≤ (signλ_2)ρ ≤ +0.035 au. Isosurface value |RGD| = 0.5 and ρ cutoff 0.04 au.

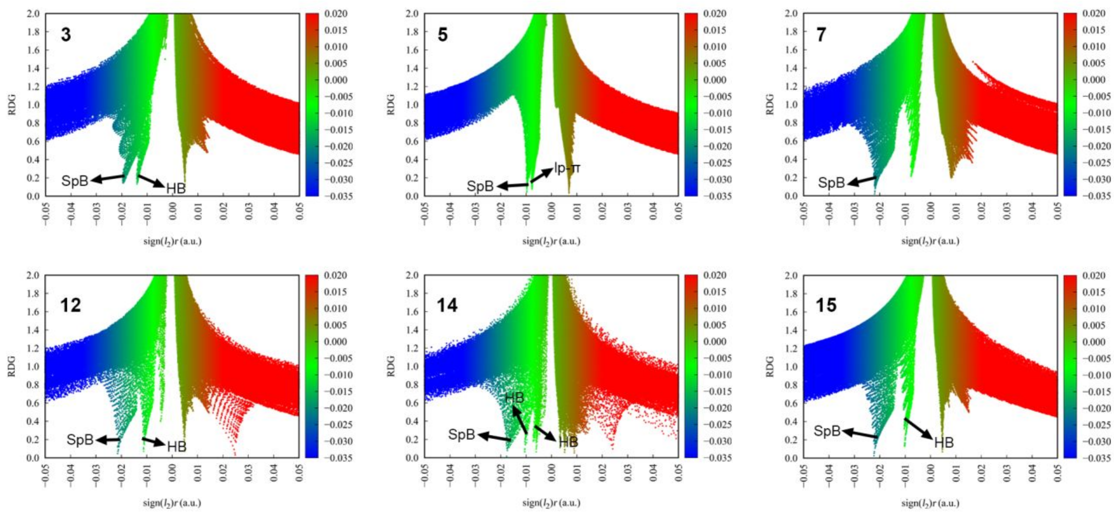

Figure S2. Graphic representation of the Reduced Density Gradient (RDG) *vs* the sign(λ_2)ρ with indication of the SpB as well as the ancillary interactions present in complexes **3**, **5**, **7**, **12**, **14** and **15**.

Cartesian coordinates of complexes 1 to 17

1.

C	2.3683736	0.8079678	0.0000000
H	2.4256336	-0.2797300	0.0000000
H	2.8627128	1.1995794	0.8889584
H	2.8627128	1.1995794	-0.8889584
Hg	0.3828243	1.3663609	0.0000000
S	-1.8405053	2.0599965	0.0000000
C	-1.6739682	3.8794888	0.0000000
H	-1.1575983	4.2354725	-0.8898899
H	-2.6844645	4.2879185	0.0000000
H	-1.1575983	4.2354725	0.8898899
C	0.4183521	-2.2412717	-1.2077401
C	-0.8110934	-1.5834419	-1.2080660
C	-1.4297058	-1.2593026	0.0000000
C	-0.8110934	-1.5834419	1.2080660
C	0.4183521	-2.2412717	1.2077401
C	1.0307488	-2.5732060	0.0000000
H	0.8955118	-2.5001083	-2.1464928
H	-1.2935572	-1.3301381	-2.1456108
H	-2.3896819	-0.7557864	0.0000000
H	-1.2935572	-1.3301381	2.1456108
H	0.8955118	-2.5001083	2.1464928
H	1.9820899	-3.0938912	0.0000000

2.

C	2.2869680	-1.5887550	0.0000000
H	1.8342205	-2.5798995	0.0000000
H	2.9078196	-1.4743041	0.8878239
H	2.9078196	-1.4743041	-0.8878239
Hg	0.7777823	-0.1841531	0.0000000
S	-0.9304940	1.3977618	0.0000000
C	-0.0071334	2.9729233	0.0000000
H	0.6114023	3.0741481	-0.8898908
H	-0.7479156	3.7720431	0.0000000
H	0.6114023	3.0741481	0.8898908
O	-1.4377285	-2.1572697	0.0000000
C	-2.5785729	-1.7396056	0.0000000
H	-3.4366511	-2.4308658	0.0000000
H	-2.7989193	-0.6618676	0.0000000

3.

C	1.2961853	-2.8073327	0.0000000
H	0.3284270	-3.3077054	0.0000000
H	1.8572940	-3.0961168	0.8878345
H	1.8572940	-3.0961168	-0.8878345
Hg	0.9504899	-0.7764840	0.0000000
S	0.5683097	1.5254623	0.0000000
C	2.2349217	2.2634917	0.0000000

H	2.7931428	1.9791354	-0.8900304
H	2.1066531	3.3454886	0.0000000
H	2.7931428	1.9791354	0.8900304
O	-1.8555733	-1.0694317	0.0000000
C	-2.8227454	-0.3192128	0.0000000
H	-3.8534425	-0.7084505	0.0000000
N	-2.7673716	1.0247459	0.0000000
H	-3.6149251	1.5646817	0.0000000
H	-1.8718024	1.4987100	0.0000000

4.

C	2.5119437	-0.9318772	0.0000000
H	2.5158251	-2.0216599	0.0000000
H	3.0300279	-0.5719638	0.8882696
H	3.0300279	-0.5719638	-0.8882696
Hg	0.5607536	-0.2611070	0.0000000
S	-1.6471621	0.4595385	0.0000000
C	-1.4624681	2.2769130	0.0000000
H	-0.9440793	2.6285118	-0.8902511
H	-2.4699534	2.6922328	0.0000000
H	-0.9440793	2.6285118	0.8902511
C	-1.5680326	-2.9398715	0.0000000
O	-2.6128033	-3.3872648	0.0000000

5.

C	0.9420458	2.5979442	0.0000000
H	2.0287381	2.5186603	0.0000000
H	0.6171945	3.1378988	0.8885901
H	0.6171945	3.1378988	-0.8885901
Hg	0.1543933	0.6937518	0.0000000
S	-0.7062167	-1.4717535	0.0000000
C	-2.5089848	-1.1857362	0.0000000
H	-2.8282133	-0.6466986	-0.8901731
H	-2.9842974	-2.1662983	0.0000000
H	-2.8282133	-0.6466986	0.8901731
N	2.9017562	-0.9299103	0.0000000
C	2.4939217	-2.0228916	0.0000000
H	2.1006814	-3.0161669	0.0000000

6.

C	2.6287100	1.4470663	0.0000000
H	2.9741859	0.4136039	0.0000000
H	3.0110641	1.9523226	0.8864997
H	3.0110641	1.9523226	-0.8864997
Hg	0.5609952	1.4628113	0.0000000
S	-1.7684144	1.6146597	0.0000000
C	-2.0651717	3.4149781	0.0000000
H	-1.6516439	3.8877931	-0.8891602
H	-3.1453045	3.5613352	0.0000000
H	-1.6516439	3.8877931	0.8891602
C	0.2640648	-3.5057306	0.0000000

C	0.9974624	-2.3421669	0.0000000
C	-1.0679568	-1.7450400	0.0000000
N	-1.0457546	-3.1021118	0.0000000
H	-1.8539830	-3.7051259	0.0000000
H	0.5480143	-4.5445113	0.0000000
H	2.0701388	-2.2319367	0.0000000
H	-1.9772046	-1.1636016	0.0000000
N	0.1613779	-1.2544611	0.0000000

7.

C	1.7241663	1.9341353	0.0000000
H	2.6818378	1.4146546	0.0000000
H	1.6582743	2.5642548	0.8864719
H	1.6582743	2.5642548	-0.8864719
Hg	0.1997700	0.5397955	0.0000000
S	-1.5656880	-0.9795065	0.0000000
C	-3.0491396	0.0823689	0.0000000
H	-3.0920218	0.7086628	-0.8893089
H	-3.9151632	-0.5793485	0.0000000
H	-3.0920218	0.7086628	0.8893089
N	1.6225980	-1.9683905	0.0000000
H	0.8145619	-2.5837498	0.0000000
H	2.1772758	-2.2028970	0.8163117
H	2.1772758	-2.2028970	-0.8163117

8.

C	2.4893232	-0.9378662	0.0000000
H	2.4884192	-2.0277463	0.0000000
H	3.0091255	-0.5809538	0.8884399
H	3.0091255	-0.5809538	-0.8884399
Hg	0.5428365	-0.2538912	0.0000000
S	-1.6625310	0.4683802	0.0000000
C	-1.4613982	2.2847826	0.0000000
H	-0.9409858	2.6328649	-0.8903516
H	-2.4659677	2.7068599	0.0000000
H	-0.9409858	2.6328649	0.8903516
O	-1.4902926	-3.0034788	0.0000000
C	-2.5766687	-3.3408625	0.0000000

9.

C	2.5453632	1.6076956	0.0000000
H	3.0716354	0.6534930	0.0000000
H	2.8241955	2.1750944	0.8873098
H	2.8241955	2.1750944	-0.8873098
Hg	0.5171440	1.2295265	0.0000000
S	-1.7706604	0.8005907	0.0000000
C	-2.4938981	2.4773563	0.0000000
H	-2.2096141	3.0363643	-0.8897026
H	-3.5766412	2.3538968	0.0000000
H	-2.2096141	3.0363643	0.8897026
C	0.0841430	-2.1428086	1.1669268

H	0.2173549	-3.2296848	1.2060602
H	0.5801861	-1.6895559	2.0235337
H	-0.9846136	-1.9074662	1.2023772
C	0.0841430	-2.1428086	-1.1669268
H	0.5801861	-1.6895559	-2.0235337
H	0.2173549	-3.2296848	-1.2060602
H	-0.9846136	-1.9074662	-1.2023772
O	0.6837535	-1.6064454	0.0000000

10.

C	2.5489893	1.4229431	0.0000000
H	2.8934427	0.3905384	0.0000000
H	2.9190527	1.9330271	0.8891258
H	2.9190527	1.9330271	-0.8891258
Hg	0.4887626	1.4264289	0.0000000
S	-1.8382393	1.5035826	0.0000000
C	-2.2137036	3.2886776	0.0000000
H	-1.8218055	3.7757820	-0.8908650
H	-3.2991154	3.3824171	0.0000000
H	-1.8218055	3.7757820	0.8908650
C	0.8905553	-1.9213864	-1.2054962
C	-0.4930244	-1.7487337	-1.2076940
C	-1.1876747	-1.6728812	0.0000000
C	-0.4930244	-1.7487337	1.2076940
C	0.8905553	-1.9213864	1.2054962
C	1.5845476	-2.0154579	0.0000000
H	1.4233095	-1.9972819	-2.1471689
H	-1.0529698	-1.6937535	-2.1344049
H	-1.0529698	-1.6937535	2.1344049
H	1.4233095	-1.9972819	2.1471689
H	2.6570329	-2.1735755	0.0000000
O	-2.5616409	-1.5955404	0.0000000
H	-2.8026369	-0.6524398	0.0000000

11.

C	2.6437009	1.7558133	0.0000000
H	2.9809222	0.7198024	0.0000000
H	3.0342106	2.2547754	0.8866589
H	3.0342106	2.2547754	-0.8866589
Hg	0.5765768	1.8257765	0.0000000
S	-1.7411290	2.0645147	0.0000000
C	-1.9551475	3.8776359	0.0000000
H	-1.5219025	4.3316860	-0.8894701
H	-3.0279761	4.0699692	0.0000000
H	-1.5219025	4.3316860	0.8894701
C	-1.1738290	-1.3802639	0.0000000
C	-1.4929221	-2.7346788	0.0000000
C	-0.4625720	-3.6689863	0.0000000
C	0.8495370	-3.2076546	0.0000000
C	1.0746284	-1.8347778	0.0000000
N	0.0886167	-0.9281184	0.0000000

H	-0.6764609	-4.7315796	0.0000000
H	-1.9504940	-0.6221419	0.0000000
H	-2.5312840	-3.0431730	0.0000000
H	1.6870902	-3.8946247	0.0000000
H	2.0861264	-1.4404360	0.0000000

12.

C	2.6532983	2.7254176	0.0000000
H	3.1448995	1.7529899	0.0000000
H	2.9458932	3.2850315	0.8877015
H	2.9458932	3.2850315	-0.8877015
Hg	0.6200768	2.3851830	0.0000000
S	-1.6860020	2.0276782	0.0000000
C	-2.3608082	3.7233129	0.0000000
H	-2.0574403	4.2723550	-0.8896925
H	-3.4469371	3.6344261	0.0000000
H	-2.0574403	4.2723550	0.8896925
C	-0.7783425	-1.4674034	0.0000000
C	-1.4477189	-2.6744451	0.0000000
C	-0.7533526	-3.8811093	0.0000000
C	0.6373128	-3.8212455	0.0000000
C	1.2908699	-2.6058517	0.0000000
N	0.5930902	-1.4247891	0.0000000
H	-1.2743863	-4.8292116	0.0000000
H	-1.2562607	-0.4972104	0.0000000
H	-2.5305805	-2.6491304	0.0000000
H	1.2387537	-4.7220759	0.0000000
H	2.3633646	-2.4727957	0.0000000
O	1.2158172	-0.3185125	0.0000000

13.

C	2.7728557	1.7438521	0.0000000
H	3.3356733	0.8111577	0.0000000
H	3.0318168	2.3209576	0.8872726
H	3.0318168	2.3209576	-0.8872726
Hg	0.7534616	1.3059815	0.0000000
S	-1.5364291	0.8722227	0.0000000
C	-2.2797378	2.5396744	0.0000000
H	-2.0016942	3.1015582	-0.8898062
H	-3.3608572	2.4015747	0.0000000
H	-2.0016942	3.1015582	0.8898062
S	0.9419071	-1.9184540	0.0000000
C	-0.1770139	-2.2740283	1.3675340
H	-0.4691393	-3.3247053	1.3558054
H	0.3615739	-2.0670673	2.2923848
H	-1.0589801	-1.6347194	1.3180785
C	-0.1770139	-2.2740283	-1.3675340
H	0.3615739	-2.0670673	-2.2923848
H	-0.4691393	-3.3247053	-1.3558054
H	-1.0589801	-1.6347194	-1.3180785

14.

C	2.5333449	0.4990138	0.2797788
H	2.5752857	-0.3626528	0.9457142
H	3.3384998	1.1928633	0.5195767
H	2.6260017	0.1608000	-0.7522789
Hg	0.6997354	1.4125591	0.4959972
S	-1.3881991	2.4433487	0.7521833
C	-1.1178475	3.5498753	2.1796355
H	-0.3496200	4.2903651	1.9637948
H	-2.0583341	4.0695075	2.3617539
H	-0.8476265	2.9932798	3.0753269
C	-0.0567385	-2.5022328	0.8447087
C	-1.0867479	-1.8769695	1.5103779
C	0.0439653	-1.8827816	-0.4358284
H	-1.4740400	-2.0475023	2.5036871
N	-1.6547340	-0.9266188	0.6962935
C	-0.9697473	-0.8850169	-0.4908733
C	0.8866140	-2.0548603	-1.5462447
H	-2.2650844	-0.1826012	1.0106896
C	-1.1427165	-0.0504738	-1.6017937
C	0.7229278	-1.2253393	-2.6441294
H	1.6580397	-2.8181239	-1.5400920
H	-1.9258483	0.7002036	-1.6288741
C	-0.2779952	-0.2329099	-2.6719964
H	1.3695148	-1.3450730	-3.5064472
H	-0.3812766	0.3948049	-3.5500562
H	0.5426268	-3.3134651	1.2290963

15.

C	1.3874004	1.6248650	0.0000000
H	2.2016459	0.9011907	0.0000000
H	1.4413114	2.2551593	0.8881231
H	1.4413114	2.2551593	-0.8881231
Hg	-0.3671095	0.5380778	0.0000000
S	-2.4388503	-0.5515587	0.0000000
C	-3.5734648	0.8805700	0.0000000
H	-3.4482897	1.5010120	-0.8874480
H	-4.5914303	0.4876428	0.0000000
H	-3.4482897	1.5010120	0.8874480
B	2.2920091	-2.2902973	0.0000000
F	2.4902768	-3.0592472	1.1495019
F	0.9522330	-1.7946766	0.0000000
F	3.1709694	-1.1896618	0.0000000
F	2.4902768	-3.0592472	-1.1495019

16.

C	1.7544112	-1.6263168	0.0000000
H	1.6349122	-2.7086022	0.0000000
H	2.3099276	-1.3149544	0.8860520
H	2.3099276	-1.3149544	-0.8860520
Hg	-0.1718122	-0.8146780	0.0000000

S	-2.0152140	0.6648676	0.0000000
C	-1.1851215	2.2918854	0.0000000
H	-0.5640645	2.4310700	-0.8856890
H	-1.9569289	3.0651411	0.0000000
H	-0.5640645	2.4310700	0.8856890
Cl	-1.5519730	-3.1045285	0.0000000

17.

C	2.2342975	-0.8600530	0.0000000
H	2.5125350	-1.9125000	0.0000000
H	2.6392327	-0.3691905	0.8863913
H	2.6392327	-0.3691905	-0.8863913
Hg	0.1478159	-0.7864825	0.0000000
S	-2.1105972	-0.1051386	0.0000000
C	-1.9438586	1.7142301	0.0000000
H	-1.4200650	2.0747080	-0.8860320
H	-2.9485577	2.1430456	0.0000000
H	-1.4200650	2.0747080	0.8860320
Br	-0.3299704	-3.6041365	0.0000000

Cartesian coordinates of the selected PDB structures

1EMS

C	1.7334746	2.0406590	-5.4186032
S	1.8014746	3.4506590	-4.2656032
C	2.2214746	-2.6653410	0.0153968
C	1.3494746	-1.7223410	0.8413968
O	1.8444746	-1.0473410	1.7503968
N	0.0604746	-1.6653410	0.4973968
C	-0.9175254	-0.8523410	1.2243968
C	-1.2645254	-1.7443410	2.4053968
O	-2.1505254	-2.5913410	2.3123968
C	-2.1725254	-0.6113410	0.3773968
C	-2.0025254	0.4236590	-0.6926032
N	-1.1595254	0.2526590	-1.7726032
C	-2.5755254	1.6416590	-0.8516032
C	-1.2215254	1.3206590	-2.5486032
N	-2.0735254	2.1776590	-2.0116032
N	-0.5525254	-1.5593410	3.5073968
C	-0.7255254	-2.3923410	4.6893968
Hg	1.4654746	2.4176590	-1.8926032
C	1.3484746	1.7486590	0.0013968
C	0.7034746	2.7846590	0.8053968
H	2.5438363	1.3150234	-5.2524578
H	0.7679922	1.5099098	-5.3958856
H	1.8547845	2.4585238	-6.4291089
H	1.2774424	3.7247666	0.8361146
H	-0.3039199	3.0395477	0.4392692
H	0.5829530	2.4712876	1.8608099

H	2.3507563	1.4821644	0.3713547
H	0.7684013	0.8178281	-0.0165100
H	-0.5641952	-1.7904020	5.5945612
H	-1.7491343	-2.7870496	4.6825516
H	-0.0250049	-3.2443683	4.6971068
H	-0.4310618	0.0830314	1.5309704
H	-2.5231796	-1.5804017	-0.0136298
H	-2.9707002	-0.2703381	1.0506393
H	-3.3027441	2.1573023	-0.2279498
H	-0.7035158	1.4460406	-3.4993662
H	0.2884054	-0.9930526	3.4273582
H	-0.3216822	-2.4256168	-0.0536477
H	2.8208225	-3.2762423	0.7033873
H	1.6555013	-3.3179101	-0.6643475
H	2.9202225	-2.0523880	-0.5731990
H	-0.5793425	-0.5659924	-1.9063577

1IRK

C	-4.7189310	0.3352712	-3.0013979
C	-3.4109310	1.0942712	-2.8423979
O	-3.0889310	1.9412712	-3.6703979
N	-2.6439310	0.7422712	-1.8143979
C	-1.3529310	1.3622712	-1.5693979
C	-0.4389310	1.0802712	-2.7533979
O	0.2500690	1.9822712	-3.2383979
C	-0.6979310	0.8242712	-0.2933979
C	0.8220690	1.0402712	-0.2793979
S	1.7140690	0.5452712	1.2196021
C	3.3490690	1.3572712	0.8746021
N	-0.4079310	-0.1667288	-3.2123979
C	0.4530690	-0.4847288	-4.3403979
C	3.9680690	-3.2347288	5.3836021
S	3.9830690	-2.4037288	3.7726021
Hg	1.7150690	-1.7877288	3.5026021
C	-0.2549310	-1.2527288	3.1736021
C	-0.5569310	0.2382712	3.2596021
H	4.9942859	-3.5802505	5.5766104
H	3.6800886	-2.5591812	6.2021900
H	3.3085174	-4.1145697	5.3950703
H	0.0588436	0.8689468	2.6202705
H	-1.6168444	0.4302143	3.0102787
H	-0.4144228	0.6077957	4.2890382
H	-0.5040144	-1.6442327	2.1708665
H	-0.8900927	-1.8098009	3.8827006
H	3.7744389	0.9954211	-0.0722482
H	3.2259351	2.4487855	0.8495882
H	4.0051521	1.0685464	1.7067631
H	1.2709649	0.4969018	-1.1249139
H	1.0312606	2.1081251	-0.4564382
H	-0.9005286	-0.2597115	-0.2135308
H	-1.1634688	1.2939269	0.5872581

H	-1.4465897	2.4581848	-1.5424944
H	-3.0301293	0.1409613	-1.0974307
H	-4.9765279	-0.3197274	-2.1560135
H	-5.5250207	1.0640711	-3.1594505
H	-4.6472339	-0.2723542	-3.9153395
H	1.4174785	-0.9064338	-4.0117542
H	-0.0417396	-1.2087943	-5.0029596
H	0.6497429	0.4449440	-4.8882702
H	-1.0579438	-0.8512825	-2.8512620

1RHY

C	-3.63386110	-3.01337930	-1.43609650
S	-3.43086110	-1.81537930	-0.13609650
C	3.93813890	-1.99537930	3.07990350
C	3.10813890	-1.69137930	1.84490350
O	3.50313890	-1.99537930	0.72990350
N	1.96013890	-1.07337930	2.05690350
C	1.07213890	-0.68737930	0.97690350
C	1.84613890	0.11962070	-0.05409650
O	1.74513890	-0.10137930	-1.26509650
C	-0.05286110	0.17162070	1.54990350
O	-0.91086110	0.62762070	0.52990350
N	2.63513890	1.06062070	0.44490350
C	3.41713890	1.93062070	-0.40509650
Hg	-3.63586110	0.12562070	-1.51909650
C	-3.86286110	1.66662070	-2.93809650
C	-4.11886110	2.94362070	-2.26809650
H	-3.53418090	-4.01332250	-0.98721540
H	-4.62868400	-2.96319570	-1.90362170
H	-2.86915100	-2.93254450	-2.22328230
H	-3.29911230	3.23960520	-1.59413360
H	-4.24067120	3.77323530	-2.98932450
H	-5.03759570	2.92938720	-1.65956780
H	-2.94097900	1.68056030	-3.54168960
H	-4.68501520	1.36335390	-3.60680910
H	-0.58557742	1.47679472	0.18975751
H	0.37336100	1.06264220	2.03879530
H	-0.60005220	-0.40663690	2.32363410
H	0.66784840	-1.56442550	0.44583640
H	1.62610070	-0.97051920	3.00714660
H	4.88924550	-1.44993610	2.99597860
H	3.45614850	-1.73051100	4.03240720
H	4.18070680	-3.06690420	3.08028420
H	4.48646550	1.66257630	-0.38785060
H	3.04687680	1.80478590	-1.43001870
H	3.30586580	2.98189120	-0.09858530
H	2.76559120	1.10421320	1.44652170

1X8K

C	-2.3753281	-2.7879626	1.0589086
C	-1.7353281	-1.6729626	0.2669086

O	-1.8003281	-0.5159626	0.6739086
N	-1.1223281	-2.0239626	-0.8620914
C	-0.5243281	-1.0509626	-1.7650914
C	3.1956719	-1.2989626	1.3289086
S	2.7346719	-0.0469626	2.6039086
Hg	1.2176719	1.9550374	1.3049086
C	0.1586719	3.8040374	0.2739086
C	0.0866719	3.5980374	-1.2250914
H	3.7548706	-2.0801771	1.8663673
H	2.3119446	-1.7623466	0.8646062
H	3.8466855	-0.8811228	0.5469778
H	1.0837673	3.5071480	-1.6814421
H	-0.4919173	2.6998148	-1.4886545
H	-0.4135069	4.4592832	-1.7052722
H	0.7862382	4.6479646	0.5896544
H	-0.8177742	3.8338531	0.7745891
H	-1.8924996	-2.8313715	2.0457799
H	-3.4294935	-2.5270033	1.2263975
H	-2.3172502	-3.7789860	0.5852308
H	0.5758039	-1.1219695	-1.7706100
H	-0.8965700	-1.1880090	-2.7915619
H	-0.8111787	-0.0546347	-1.4033942
H	-1.1037689	-2.9989138	-1.1263731

3PYK

C	-0.85556210	-2.50610520	-2.38267570
S	0.76943790	-2.01910520	-2.98967570
Hg	1.70243790	-0.84110520	-1.27567570
C	2.62043790	0.29989480	0.15332430
H	-0.79777400	-3.38657390	-1.72538020
H	-1.35153520	-1.68521490	-1.84870900
H	-1.45862480	-2.77520920	-3.26205480
H	2.94636550	1.26565580	-0.26532630
H	1.92968770	0.50887180	0.98595970
H	3.50879400	-0.19980550	0.56961170
C	-0.47356210	2.42189480	-0.17767570
C	-0.78056210	1.01889480	0.34432430
O	-1.05956210	0.11589480	-0.43567570
N	-0.75156210	0.84489480	1.67632430
C	-1.00756210	-0.47810520	2.22032430
H	0.52153540	2.40469130	-0.64764240
H	-1.20672320	2.65996530	-0.95873760
H	-0.48719340	3.19886600	0.59984100
H	-0.07208440	-1.03542760	2.39817610
H	-1.55949220	-0.40162220	3.16773270
H	-1.60521730	-1.03488100	1.48890180
H	-0.47769990	1.60393310	2.28380410

5LU8 (TYR220)

C	3.9446024	-0.7078690	-0.5999027
S	2.8066024	0.2341310	-1.6329027

C	-0.4223976	-2.1868690	-1.6429027
C	-0.7933976	-2.3298690	-0.3019027
C	-0.9333976	-1.1038690	-2.3529027
C	-1.6053976	-1.4008690	0.3150973
C	-1.7583976	-0.1758690	-1.7499027
C	-2.0923976	-0.3308690	-0.4139027
O	-2.9223976	0.5781310	0.1840973
Hg	1.3436024	0.9311310	0.2750973
C	0.0346024	1.4031310	2.1790973
C	-0.3663976	2.8591310	2.1020973
H	4.5006698	-0.0758084	0.1086115
H	3.4441650	-1.5141357	-0.0422219
H	4.6820181	-1.1806899	-1.2671616
H	0.5027508	3.5333378	2.0701850
H	-0.9825160	3.0593150	1.2125490
H	-0.9709813	3.1521586	2.9808865
H	0.6759645	1.1494366	3.0335807
H	-0.8074457	0.7051901	2.0939451
H	-1.8982391	-1.5060549	1.3616799
H	-0.4176073	-3.1776261	0.2772698
H	0.2454607	-2.9036858	-2.1217634
H	-0.6543221	-0.9688865	-3.4010048
H	-2.1486451	0.6737800	-2.3185575
H	-3.1636370	1.2470840	-0.4640860

5LU8 (TRP232)

C	-0.0945731	-3.1311706	-0.8069025
S	-1.2325731	-2.1891706	-1.8399025
C	1.0024269	-0.4461706	-4.4549025
C	0.5134269	0.4998294	-3.3619025
O	-0.4695731	1.2228294	-3.5619025
N	1.1974269	0.4958294	-2.2139025
C	0.7294269	1.2158294	-1.0159025
C	0.3954269	2.6778294	-1.2629025
O	-0.6775731	3.1498294	-0.8939025
C	1.7644269	1.1378294	0.1150975
C	1.7754269	-0.1601706	0.8090975
C	2.6144269	-1.2051706	0.5800975
C	0.8964269	-0.5681706	1.8550975
N	2.3144269	-2.2461706	1.4200975
C	1.2604269	-1.8821706	2.2150975
C	-0.1695731	0.0478294	2.5250975
C	0.5954269	-2.5961706	3.2180975
C	-0.8285731	-0.6611706	3.5270975
C	-0.4445731	-1.9701706	3.8590975
N	1.3254269	3.4058294	-1.8629025
C	1.1274269	4.8388294	-2.0989025
Hg	-2.6955731	-1.4921706	0.0680975
C	-4.0045731	-1.0201706	1.9720975
C	-4.4055731	0.4358294	1.8950975
H	-3.5307791	1.1033099	1.8677896

H	-5.0118494	0.6516391	1.0030716
H	-5.0098442	0.7275341	2.7745489
H	-3.3360546	-1.2745410	2.8026201
H	-4.8449385	-1.7252799	1.9171096
H	0.2774880	-2.5444333	0.0399486
H	-0.5416918	-4.0622029	-0.4257355
H	0.7720258	-3.4089425	-1.4279525
H	2.0481089	-0.7658050	-4.3313349
H	0.3523783	-1.3347080	-4.4374693
H	0.8745164	0.0554652	-5.4218081
H	1.8161272	-0.2865464	-2.0350707
H	-0.2229762	0.7737325	-0.6795769
H	2.0928875	5.3618549	-2.0530362
H	0.6560211	5.0263601	-3.0776434
H	0.4555939	5.2174781	-1.3191014
H	2.1035513	2.9320460	-2.3032407
H	2.7597798	1.3813384	-0.2915700
H	1.5227041	1.9345694	0.8359550
H	3.4471373	-1.2798985	-0.1187119
H	0.8932494	-3.6148050	3.4776568
H	-0.9846649	-2.4988986	4.6484188
H	-1.6521394	-0.1877625	4.0657253
H	-0.4802743	1.0626659	2.2620184
H	2.7877722	-3.1366682	1.4459295

6BZI (THR375)

C	0.3080768	-3.4151683	2.2681550
C	-0.5679232	-2.4321683	1.4861550
O	-1.7869232	-2.4581683	1.6071550
N	0.0680768	-1.5771683	0.6841550
C	-0.6399232	-0.6481683	-0.2008450
C	-1.6129232	-1.3741683	-1.1288450
O	-2.7609232	-0.9431683	-1.2768450
C	0.3390768	0.1998317	-1.0408450
O	1.2061098	0.9206305	-0.1612980
C	-0.3969232	1.1818317	-1.9228450
N	-1.1609232	-2.4711683	-1.7368450
C	-2.0339232	-3.2741683	-2.5998450
C	3.0490768	4.5168317	-1.2448450
S	3.6030768	4.0228317	0.4141550
Hg	1.3150768	3.4918317	1.3041550
C	-0.6189232	2.3828317	2.0621550
C	-0.2019232	1.2238317	2.9361550
H	3.9453200	4.6448713	-1.8709624
H	2.4119677	3.7598566	-1.7288822
H	2.5085078	5.4746111	-1.2354750
H	0.2536329	1.5591718	3.8798143
H	-1.0780857	0.6006378	3.1942504
H	0.5154492	0.5693820	2.4203739
H	-1.0853725	2.0842362	1.1152133
H	-1.2468329	3.1251316	2.5723013

H	1.3663551	-3.1220372	2.3301343
H	-0.1109043	-3.5148863	3.2770410
H	0.2429277	-4.3993857	1.7801386
H	1.0679656	-1.4441379	0.7754087
H	-1.2806681	0.0196876	0.3948751
H	-1.5020583	-4.1869464	-2.8953545
H	-2.9583963	-3.5383224	-2.0647395
H	-2.3128227	-2.7074531	-3.5003953
H	-0.2703976	-2.8498278	-1.4425609
H	0.3268331	1.7955152	-2.4810783
H	-1.0578065	0.6851040	-2.6467667
H	-1.0157338	1.8500058	-1.3065287
H	0.9372386	-0.4970749	-1.6612032
H	1.9910719	1.2033404	-0.6454704

6BZI (GLU448)

C	1.4493109	0.9065575	-0.1094111
S	2.0033109	0.4125575	1.5495889
C	-3.4376891	8.3655575	-0.7704111
C	-3.5176891	7.2395575	0.2615889
O	-4.5626891	6.6145575	0.4085889
N	-2.4096891	6.9545575	0.9405889
C	-2.3816891	5.8675575	1.9245889
C	-3.2906891	6.1665575	3.1215889
O	-3.8586891	5.2475575	3.7155889
C	-0.9506891	5.5705575	2.3835889
C	-0.0166891	5.0715575	1.2835889
C	-0.5266891	3.8325575	0.5585889
O	-1.0866891	2.9325575	1.2265889
O	-0.3576891	3.7635575	-0.6804111
N	-3.4346891	7.4515575	3.4505889
C	-4.4446891	7.9135575	4.4035889
Hg	-0.2846891	-0.1184425	2.4395889
C	-2.2186891	-1.2274425	3.1975889
C	-1.8016891	-2.3864425	4.0715889
H	-4.0139771	8.6280877	5.1225265
H	-5.2966464	8.3864117	3.8870334
H	-4.8035760	7.0245498	4.9388929
H	-3.0273513	8.1245450	2.8138027
H	-2.7938962	4.9484612	1.4803495
H	-0.5362974	6.4568720	2.9014344
H	-1.0476767	4.7597510	3.1215422
H	0.1919862	5.8459019	0.5249473
H	0.9535169	4.8154327	1.7431041
H	-1.5201131	7.3250208	0.6301009
H	-2.5160408	8.3088189	-1.3702780
H	-4.3119524	8.2783592	-1.4248755
H	-3.4595109	9.3487033	-0.2728035
H	-1.2549642	-2.0516461	4.9673134
H	-2.6756524	-2.9663256	4.4283230
H	-1.1460172	-3.0928658	3.5377833

H	-2.7276430	-1.5307620	2.2718383
H	-2.8209829	-0.4686506	3.7149196
H	2.2971940	1.4109420	-0.5984628
H	1.1750200	0.0326040	-0.7204583
H	0.6112064	1.6237589	-0.0899956

6PII

C	1.7852011	-1.7653795	1.5334566
C	0.8432011	-0.5843795	1.3474566
O	0.5352011	-0.1603795	0.1714566
N	0.4292011	0.0256205	2.4564566
C	-0.3117989	1.3016205	2.4154566
C	1.9792011	2.0476205	-2.8265434
S	0.4972011	2.8896205	-2.2355434
Hg	-1.0627989	1.1846205	-1.7945434
C	-2.4347989	-0.3983795	-0.9115434
C	-2.1627989	-1.7443795	-1.3195434
H	2.7841069	2.7974758	-2.8456336
H	2.2841726	1.2304666	-2.1552197
H	1.8574783	1.6510744	-3.8453759
H	-1.1183962	-2.0362382	-1.1191227
H	-2.7983676	-2.4773765	-0.7836673
H	-2.3395648	-1.9188432	-2.3939898
H	-2.2441064	-0.2266253	0.1594623
H	-3.4665341	-0.0770921	-1.1256216
H	2.7945846	-1.4422205	1.2381022
H	1.8163172	-2.1448340	2.5647298
H	1.4845946	-2.5719174	0.8519674
H	-0.0288466	1.8336315	1.4967810
H	-1.4003188	1.1345907	2.4038685
H	-0.0462114	1.9035329	3.2944983
H	0.6954112	-0.3514874	3.3555072

1L9A

C	0.6772106	4.0806567	5.2847924
C	-0.0837894	3.3326567	4.1867924
O	-1.3127894	3.2896567	4.2157924
N	0.6492106	2.7496567	3.2347924
C	0.0742106	2.0506567	2.0747924
C	-1.2707894	1.3676567	2.3137924
O	-1.4637894	0.6746567	3.3147924
C	1.0492106	0.9906567	1.5607924
C	0.6222106	0.3436567	0.2627924
C	0.6972106	1.0336567	-0.9422076
C	0.1482106	-0.9603433	0.2397924
C	0.3132106	0.4406567	-2.1332076
C	-0.2357894	-1.5613433	-0.9432076
C	-0.1557894	-0.8553433	-2.1262076
O	-0.5407894	-1.4473433	-3.3062076
N	-2.1867894	1.5576567	1.3717924
C	-3.4687894	0.8746567	1.3947924

C	-0.0587894	-6.3903433	-4.2432076
S	0.6852106	-5.2513433	-5.4372076
Hg	1.9322106	-3.3043433	-4.9222076
C	2.5932106	-1.6243433	-4.0072076
H	0.6900254	-7.0045267	-3.7221881
H	-0.6938358	-5.8859330	-3.5000658
H	-0.6878355	-7.0444422	-4.8616552
H	3.6817163	-1.4760834	-4.0673372
H	2.1102752	-0.7162486	-4.3985712
H	2.3112607	-1.7361461	-2.9491191
H	-0.4343322	-0.8126884	-4.0215269
H	-0.6110174	-2.5864346	-0.9638072
H	0.0686668	-1.5184125	1.1764319
H	0.3859871	0.9957386	-3.0731821
H	1.0818360	2.0582231	-0.9564691
H	2.0409560	1.4582477	1.4242540
H	1.1522289	0.2287459	2.3491530
H	1.6578145	2.7779635	3.2899856
H	1.7588977	4.1761054	5.1084710
H	0.2302189	5.0791192	5.3787511
H	0.5119658	3.5538403	6.2356717
H	-3.5096252	0.3008367	2.3289135
H	-4.2993478	1.5969416	1.3803949
H	-3.5700017	0.1882403	0.5388768
H	-1.9586028	2.1327712	0.5729397
H	-0.0667369	2.7986937	1.2691185

2D2N

C	-0.0467956	-4.2994972	8.1120000
S	1.1832044	-2.9664972	8.1180000
Hg	3.2472044	-3.0674972	6.3570000
C	5.8152044	-3.4134972	5.7340000
H	-0.7276109	-4.0598073	8.9438458
H	-0.6359399	-4.3322533	7.1834513
H	4.6411726	-0.3652509	8.4627262
H	6.2629903	-3.0114642	6.6468131
H	5.9249966	-4.4920682	5.5944809
H	5.9422893	-2.8076630	4.8336167
C	2.6142044	0.2265028	5.1860000
C	2.0072044	0.3625028	6.4310000
C	3.9562044	-0.1324972	5.1340000
C	2.7282044	0.1545028	7.6080000
C	4.6882044	-0.3414972	6.3060000
C	4.0742044	-0.1964972	7.5440000
H	0.3914141	-5.2907028	8.3000417
H	2.2260485	0.2397514	8.5735755
H	5.7413449	-0.6192764	6.2432687
H	4.4504541	-0.2538323	4.1662476
H	0.9466115	0.6167504	6.4949491
H	2.0440927	0.3853757	4.2682373

5G5N

C	0.9571154	0.4567176	2.2185445
C	0.2731154	-0.5362824	1.3245445
O	0.2791154	-0.3552824	0.1215445
N	-0.3018846	-1.5972824	1.8765445
C	-0.9678846	-2.5682824	1.0135445
C	-0.3758846	-0.8972824	-3.2494555
S	-0.8248846	0.8527176	-3.2534555
Hg	-0.5138846	1.8617176	-1.7264555
C	0.5401154	3.4597176	-0.4364555
H	0.8275389	4.2762681	-1.1146991
H	-0.0526377	3.8635821	0.3959055
H	1.4437922	2.9662300	-0.0489141
H	-0.0255393	-1.2165243	-2.2575116
H	-1.2486814	-1.4988200	-3.5399819
H	0.4251090	-1.0485346	-3.9860161
H	2.0179452	0.4997232	1.9340529
H	0.5326604	1.4505930	2.0169146
H	0.8807019	0.2388074	3.2937961
H	-0.2430595	-3.0756526	0.3581879
H	-1.4773270	-3.3159628	1.6344120
H	-1.7044472	-2.0634178	0.3715418
H	-0.2815914	-1.7351234	2.8765731
